# Maternal and child health voucher scheme in Myanmar: a review of early stage implementation

**DOI:** 10.1186/s12913-016-1850-3

**Published:** 2016-10-21

**Authors:** Songyot Pilasant, Wantanee Kulpeng, Pitsaphun Werayingyong, Nattha Tritasavit, Inthira Yamabhai, Yot Teerawattananon, Sangay Wangmo, Sripen Tantivess

**Affiliations:** 1 Health Intervention and Technology Assessment Program (HITAP), Ministry of Public Health, Tiwanon Road, Muang, Nonthaburi Thailand; 2 International Health Policy Program (IHPP), Ministry of Public Health, Tiwanon Road, Muang, Nonthaburi Thailand

**Keywords:** Maternal and child health, Evaluation

## Abstract

**Background:**

The Maternal and Child Health Voucher Scheme (MCHVS) was introduced in Myanmar to address the high rate of maternal and infant mortalities. It aimed to increase access to maternal and child health (MCH) services by skilled birth attendants (SBAs) and improve the health of pregnant women and their babies. A study to pilot a voucher scheme was implemented in May 2013 in Yedarshey Township. This paper provides a report on a mid-term review of the programme after 7 months of implementation to determine the outcomes of the programme and its impediments.

**Methods:**

Quantitative and qualitative approaches were used. Secondary quantitative data were analysed in order to measure the coverage and utilisation of the programme. Semi-structured interviews were conducted in groups and individually with 79 key informants to explore qualitative information on voucher communication, beneficiary’s identification, voucher distribution, and challenges for beneficiaries and providers under the MCHVS.

**Results:**

The results showed that 63 % of eligible pregnant women who registered to the programme received voucher booklets, while the utilisation of most of the MCH services increased over time; in particular, delivery by SBAs increased significantly (*P* < 0.01) after implementing MCHVS. Overall, the programme was implemented well in terms of promoting and communicating the programme to people in Yedarshey Township. Although a number of targeted poor pregnant women were included in the programme, some beneficiaries were overlooked for a variety of reasons. Nevertheless, both providers and beneficiaries who experienced the MCHVS service utilisation were satisfied with the programme. The evaluation indicated several programme challenges, i.e. external and internal programme communication, voluntary voucher distributor recruitment, incentive and support for voucher distributors, beneficiary screening criteria, and approaches to increase access of services for pregnant women living in remote areas.

**Conclusions:**

Generally, the MCHVS pilot programme is a promising initiative to increase access to and utilisation of the MCH services for pregnant women and their babies in Myanmar. However, increasing coverage of the programme and overcoming the barriers should be considered as high-priority issues that need to be addressed.

## Background

Myanmar is a country in Southeast Asia where a majority of the population (70 %) live in rural areas [1] and 25.6 % of the population lived below the national poverty line [2]. Myanmar faces many challenges in implementing health interventions, especially strongly encouraging pregnant women, mothers, and children who live in rural remote areas to utilise maternal and child health (MCH) services [3]. Although maternal and child mortality rates have improved steadily in the last decade [4], it is still high compared to other countries in the region. Myanmar had a low rate of MCH services utilisation, especially in rural areas. Only 67.6 % of pregnant women in rural areas had four visits of antenatal care (ANC) [5], which is the minimum care recommended by the World Health Organization (WHO) [6]. In terms of delivery, less than one-fourth (24.5 %) of pregnant women who lived in rural areas delivered their babies at health facilities. Only 40 and 19.7 % of those who lived in urban and rural areas, respectively, delivered their babies at home with assistance from skilled birth attendants (SBAs) [7]. For postnatal care, just over half of new mothers (56.6 %) received care from at least one visit [7]. The immunisation program for babies seemed to be successful as a MCH service in Myanmar, where 88 and 90 % of children were given the measles vaccine and diphtheria, pertussis, and tetanus (DPT) vaccines, respectively [8]. Regarding health outcomes, under-five mortality rate and maternal morlity ratio were 56 per 1,000 live births and 220 per 100,000 live births, respectively [9]. Although the mortality trends decreased, the mortality rate was still far from achieving the Millennium Development Goal (MDG) 4 in reducing under-five mortality rate to 36 per 1,000 live births [10] and MDG 5 in reducing maternal mortality ratio to 130 per 100,000 live births [11].

One major barrier that prevented pregnant women from accessing MCH services, resulting in low service utilisation, was the distance between homes and health facilities, as well as a lack of transportation [12]. However, distance is not the only factor that prevents them from accessing MCH services as financial difficulties also play a role [7, 13]. Evidence shows that 39 % of pregnant women had to borrow money for MCH services, costing 147 PPP US$ (32,000 kyats) and 129 PPP US$ (28,000 kyats) for delivery assisted by SBAs and non-SBAs, respectively [14]. Each figure accounted for more than one-sixth of Myanmar’s gross domestic product (GDP) per capita [15] and was higher than the national poverty line (4.7 PPP US$ or 1030 kyats per day) by approximately 30 times, and also contributed to almost 20 % of loan size of the poor [16].

### The development and the pilot of maternal and child health voucher scheme in Myanmar

Demand-side financing is an intervention to improve access to and utilisation of health services, especially among the poor [17]. For MCH services, demand-side financing was implemented in various forms such as conditional cash transfer and a voucher programme. In 2010, the WHO and Ministry of Health (MoH), Myanmar in collaboration with the Health Intervention and Technology Assessment Program, Thailand, conducted a study in order to determine an appropriate design for a programme to reduce financial barriers to government-provided MCH services [18]. The study suggested that demand-side financing, particularly vouchers, was feasible and cost-effective in the Myanmar context and financial subsidisation should be given for both providers and clients in order to address the financial difficulties that prevented beneficiaries from accessing MCH services. The research also offered contextually-relevant recommendations on the design and implementation of the voucher programme. These included, for example, voucher distributors and distribution channels, communication strategy, services package, and subsidisation. These recommendations were collated into a set of guidelines for programme implementation in 4 main areas, which include voucher distribution, voucher communication, financial management of the programme, and programme monitoring and evaluation.

The pilot demand-side financing programme – MCH voucher scheme (MCHVS) – was introduced in Yedarshey Township, Nay Pyi Taw in May 2013. It aimed to increase MCH services of low-income pregnant women and newborns; hence, activities in this pilot programme were focused on services for pregnant women and newborns only, namely antenatal care (ANC), delivery, postnatal care (PNC), and infant-immunisation.

There were many steps in this programme. Firstly, in order to ensure that the vouchers reached the target group, various groups of people, such as community health volunteers, local authorities, shopkeepers, and school teachers were recruited to be voucher distributors. The diversity of voucher distributors would complement voucher distribution by midwives (MWs) because MWs worked full-time at health facilities and, thus, it was unlikely that they could meet with and give vouchers to pregnant women who had limited access to facility-based services. Meanwhile, poor pregnant women who were eligible for the programme were sought and identified by community leaders. In doing so, the community leaders had to follow criteria, which indicated that the pregnant women in households that possessed tractors, motorcycles, or mobile phones were excluded; on the other hand, those who faced severe financial issues and needed to seek support for travelling and other basic necessities, and also had daily household income less than 4.6 PPP US$ (1,000 kyats) were eligible.

Eligible pregnant women had to register to the programmers for the MoH to estimate the number of voucher booklets that should be distributed. Each registered pregnant woman would receive one voucher booklet. Each booklet contained coupons that covered 4 services: ANC, delivery, PNC and infant- immunisation. These women could select to receive care, including delivery, either at health facilities or at home, except for the first ANC which was provided only at health facilities. The pregnant women had to inform their preference for where to receive care to midwives in the first ANC visit. All care was provided to pregnant women by SBAs regardless of setting. In addition to utilising these free services, pregnant women could use these coupons to reimburse travel, food, and accommodation costs when receiving care at health facilities. These coupons were considered financial incentives for the beneficiaries and were valued at 27.6 PPP US$ (6,000 kyats) for services at home and 64 PPP US$ (14,000 kyats) for services at health facilities [19].

After 7 months of implementing the programme, the MoH requested a programme evaluation in order to identify obstacles that needed to be addressed at the early stage of implementation. As the evaluation was conducted during the early stage of programme implementation, it was focused more on the process and primary outcomes of the programme. The findings of the evaluation were categorized into five main themes, namely voucher coverage, voucher utilisation, programme communication, identification of beneficiaries and voucher distribution, and challenges for beneficiaries and providers under the MCHVS.

## Methods

### Study design

Both quantitative and qualitative approaches were employed in this study. Quantitative methods were used to measure coverage and utilisation of the programme, while semi-structured interviews (SSIs) were conducted to explore the qualitative data on voucher communication, beneficiary’s identification, voucher distribution, and challenges for beneficiaries and providers under the MCHVS.

### Quantitative methods

#### Data collection

To measure the coverage and utilisation outcomes of the programme compared to before the pilot’s launch, data collection forms were developed to obtain secondary data on the services used. The forms were filled by local persons who were responsible for programme management. The data, including name and level of health facilities, monthly incidence of pregnancy cases, number of vouchers distributed to registered pregnant women, number of pregnant women coming to receive the first ANC, delivery, and PNC by SBAs, and number of infants who had been vaccinated, between January and December 2013 were collected from all health facilities in Yedarshey Township; these facilities include one maternal and child health clinic and eight rural health centres (RHCs).

#### Data analysis

Descriptive statistics were used to illustrate the voucher distribution and its coverage by each RHC in Yedarshey Township from June to December 2013. The voucher coverage was calculated as a percentage by dividing the number of vouchers distributed to pregnant women over the total number of registered women in each RHC.

### Qualitative methods

Group and individual SSIs were performed with 79 key informants, which consisted of (1) SBAs, i.e. doctors, nurses, lady health visitors, midwives (MWs), and a health assistant; (2) non-SBAs, i.e. auxiliary midwives (AMWs) and traditional birth attendants (TBAs); (3) beneficiaries, i.e. pregnant women, new mothers, and their relatives; (4) villagers; and (5) MoH and WHO staff. The evaluators suggested that these five groups of key informants should be interviewed. The MoH staff were responsible for selecting certain areas for the group SSIs, which consisted of three villages in Yedarshey, namely Aung Chan Thar, Amagyikhone, and Swar, and five health facilities, namely Yedarshey Township hospital, Aung Chan Thar RHC, Swar station health unit, and Ga Ra sub-centre. Convenience sampling was used by the MoH staff to recruit all key informants.

A list of questions for both group and individual SSIs was developed and translated to the Myanmar language by the evaluators and the translated version of these questions was sent to MoH staff beforehand. Individual SSIs were conducted with MoH and WHO staff in English by the evaluators at the MoH. The group SSIs were carried out with other groups, apart from MoH and WHO staff, which were separated into nine groups: three groups of SBAs, two groups of non-SBAs, and four groups of beneficiaries and villagers (Table 1). To conduct the group SSIs, the evaluators were separated into two teams in order to triangulate the data. Each team performed four sessions of SSIs at the Station Health Unit, Rural Health Centre, and Sub-Centre, except for the group interview with SBAs at the Township hospital, which the evaluators conducted together. The questions for group SSIs were asked in Myanmar and translated into English for the evaluators by the MoH and WHO staff. All SSIs were conducted on 22 and 23 January 2014.

**Table 1:** Characteristics, number of, and interview techniques used with key informants

Category	Key informants	Number of key informants				Qualitative techniques
		Township hospital	Station health unit	Rural health centre	Sub-centre	
Skilled birth attendants	Doctors	2	-	-	-	Group semi-structured interviews (SSIs) in Myanmar language with English interpreters
	Nurses	2	-	-	-	
	Midwives	2	2	4	1	
	Lady health visitors	1	1	1		
	Health assistant	1	-	-	-	
Non-skilled birth attendants	Auxiliary midwives	-	3	-	3	
	Traditional birth attendants	-	-	-	1	
Beneficiaries	Pregnant women	-	21	3	-	
	New mothers	-	3	7	-	
	Relatives	-	-	2	-	
Villagers	Villagers	-	11	1		
Ministry of Health (MoH) and World Health Organization (WHO) staff ^a^	Staff from MoH	-	2	2		Individual SSIs in English
	Staff from WHO	-	1	2		

^a^Interviews with staff from the MoH and WHO were performed at the MoH

## Results

### Voucher coverage

The number of pregnant women who registered for the pilot programme in nine facilities between June and October 2013 (Table 2) amounted to 2,137. A total of 1,800 vouchers were distributed to these facilities according to the number of pregnant women who had sought MCH care at those locations. During the same period, 1,346 registered women received the vouchers. The voucher coverage in these areas varied from 40 to 95 %, where the highest coverage was found in Mayokhone, the lowest-income area in Yedarshey Township. Yae Ne, the second highest-income area, had the highest number of pregnant women registered but only 40 % of them received voucher booklets, which was the lowest coverage among all areas. The interviews with key informants revealed that the low amount of voucher distributions in some areas might be because of rumours that the programme would be terminated in September 2013. Hence, recruitment of eligible pregnant women decreased and voucher distributors were reluctant to provide the pregnant women with vouchers due to concerns about the program’s discontinuation.

**Table 2:** Voucher coverage from June to October 2013 by setting (ordered by economic status – richest to poorest – of the area that each setting covered)

Economic status (richest to poorest)	Setting	Number of women registered (A)	No. of vouchers distributed to the setting (B)	No. of vouchers distributed to target pregnant women (C)	Percent coverage (% of C/A)
1	Swar RHC	340	310	248	73
2	Yae Ne RHC	411	230	163	40
3	MyoHla RHC	297	210	181	61
4	Maternal and Child Health clinic	84	134	68	81
5	Amagyikhone RHC	258	243	217	84
6	Aung Chan Thar RHC	250	183	112	45
7	Kyar Inn Kone RHC	276	200	170	62
8	HlaePyawe Lay RHC	69	120	42	61
9	Mayokhone RHC	152	170	145	95
Total		2,137	1,800	1,346	63

### Voucher utilisation

**Fig. 1:**
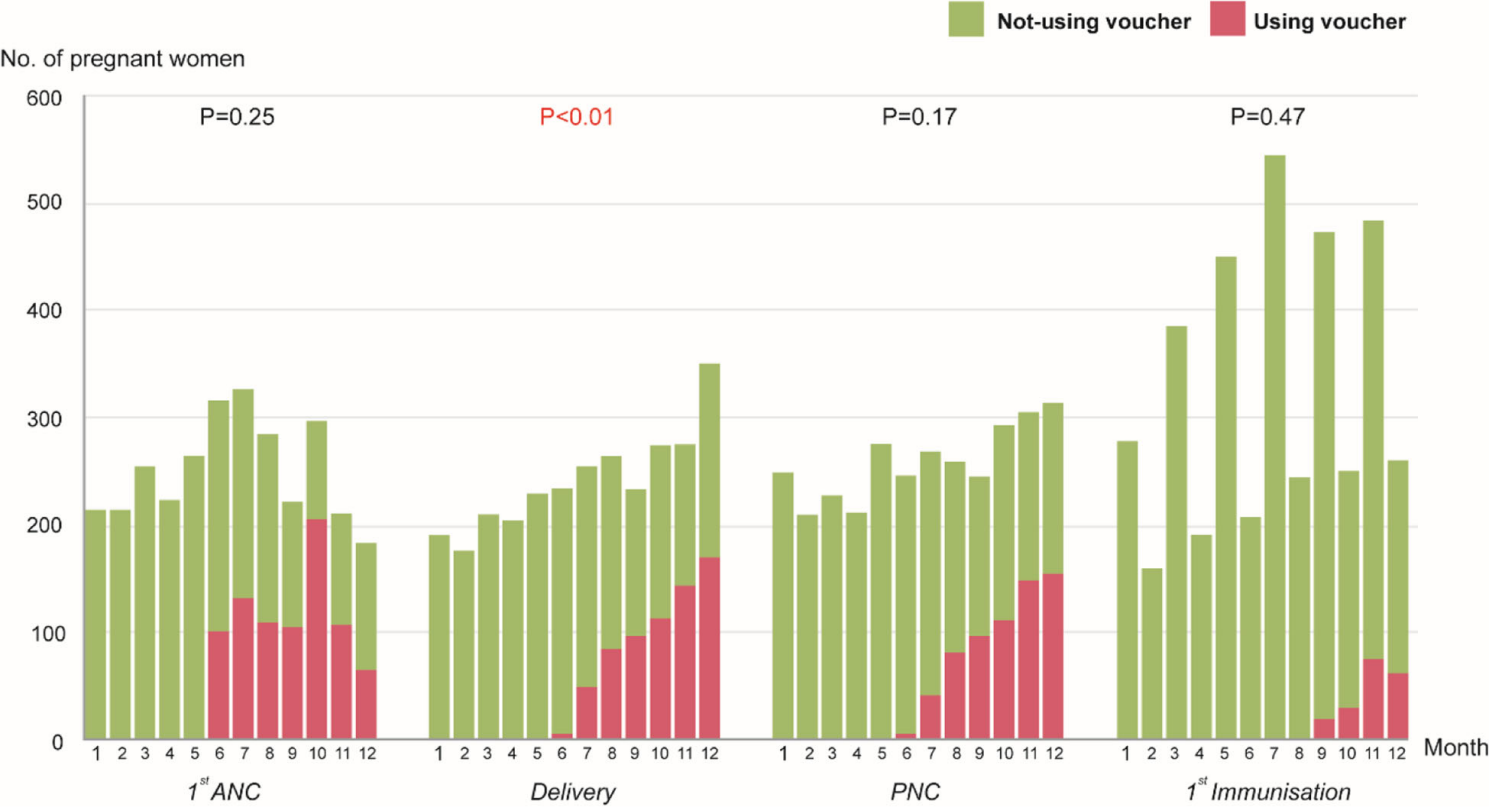
Number of monthly clients of MCH services in Yedarshey Township, pre- and post-implementation of the voucher programme (January to May and June to December 2013, respectively)

Figure 1 shows that the number of pregnant women receiving MCH services in the voucher scheme increased over time, except for the first ANC. The number of women who sought the first ANC increased during the first half of the year and declined during the second half. Although the number of deliveries by SBAs increased over the year, it significantly increased after the implementation of the MCHVS (*P* < 0.01). Similarly, PNC visits and immunisations also increased after programme implementation but the increase was statistically insignificant. For infant’s first immunisation, the data showed irregular increases in numbers during alternating months due to the immunisation schedule of some facilities.

**Fig. 2:**
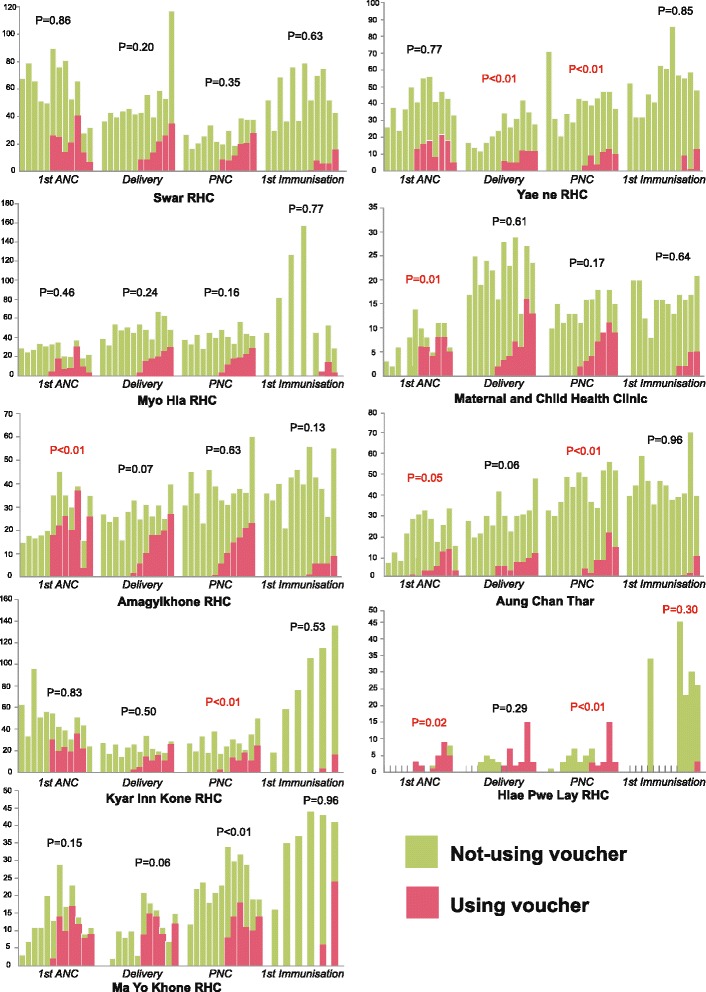
Number of monthly clients by services in nine settings of Yedarshey Township, pre- and post-implementation of the voucher programme in January to May and June to December of 2013

Figure 2 was disaggregated from Fig. 1, representing a sub-group analysis (the nine areas in Yedarshey Township) in order to show the variation in terms of access to and utilisation of MCH services among poor pregnant women across the nine areas. It can be seen that the number of first ANC visits, deliveries by SBAs, and PNC visits increased in some areas after implementation. There were two areas, namely Swar and Myo Hla, with insignificant increases in MCH services after the implementation of the MCHVS. Although Aung Chan Thar and Yae Ne had low coverage of the MCHVS compared to other areas, these two areas still observed significant increases in ANC and PNC, and deliveries by SBAs and PNC, respectively. Aung Chan Thar also observed a significant increase in deliveries by SBAs. In the remaining five areas, there was at least one type of MCH service utilisation that increased significantly after the implementation of the programme during the second half of the year.

For the fluctuating trends of immunisation services used in some areas, interviews with the MoH staff indicated that there were other immunisation programmes provided by international organizations which were not afforded to the MCHVS’s beneficiaries every month, especially those who lived in remote areas and mountainous terrains; instead, it was provided to them every 6 weeks. Additionally, increasing recognition of the voucher programme led to an overall increasing trend in immunisation.

### Programme communication

The study found that various techniques were used to communicate the scheme to communities. These techniques included posters, community loudspeakers, events, and some advertisements printed on accessories for voucher distributors, such as umbrellas, waistcoats, and bags. These media were illustrated with the programme’s title in the local language in order to maximize exposure to the target groups and other villagers. These media, especially posters, were widely recognised by people in communities. The posters were normally seen at the RHCs as well as many places in villages, such as markets and schools. In terms of effectiveness, personal communication between voucher distributors and pregnant women seemed to be a communication channel that mostly gave information about the programme to the pregnant women and led them to utilise services. Moreover, it was found from the group interviews with villagers that men played an important role in the dissemination of information in the communities, which can be another effective strategy as men are more likely to gather together than women.

More importantly, the message about the expected health outcomes of mothers and infants did not reach the communities and instead focused solely on the financial benefits provided. The interviews also showed that most of the pregnant women in the villages joined the programme simply because the voucher distributors recommended them to do so, and thus they sought out care from SBAs at the health facilities without any awareness and expectation for desirable health outcomes. However, it was found from the interviews that there were some pregnant women and new mothers who did not know about the scheme; they revealed that no information regarding this programme had ever been sent to them.

### Beneficiary’s identification and voucher distribution

Although the MCHVS targeted pregnant women who were poor and lived in remote areas, it was rather difficult to identify and recruit eligible ones. This problem was caused by the criteria used in the scheme. It signified a grey zone, considering that some pregnant women who had difficulties covering the cost of ANC and delivery were neglected. These criteria, such as daily maximum income, were not as helpful as expected due to seasonal fluctuations in household income from those in the agriculture sector, or pregnant women whose families had many members and earned slightly higher than the cut-off point.

In urban areas, identifying the poor and enrolling them in the MCHVS was more difficult than the rural counterparts. This was because people residing in urban areas felt considerably stigmatised when they were classified as poorer than others. In rural areas, this might not be the case since most people were in the same income group and eligible for the voucher scheme benefits. The interviews with MWs who were voucher distributors revealed that stigmatisation was prevalent, especially in Swar – the highest economic status location – even though the health personnel responsible for the communications campaigns put tremendous effort to avoid this undesirable consequence.

In the beginning of the programme, most voucher distributors were MWs because the programme faced difficulties in recruiting and training non-MWs to be voucher distributors. In particular, compared to MWs, it took a longer time to recruit and train non-MWs which resulted in insufficient time to complete training on time. Although the concept of the programme was to distribute vouchers on a voluntary basis, non-MW voucher distributors still wanted support in various forms for distributing vouchers to the beneficiaries, e.g. bicycles for travelling to beneficiaries’ houses, financial support for communication with the MWs, and financial incentives.

### Challenges for beneficiaries and providers under the MCHVS

It was found that pregnant women who had experiences with using vouchers and services provided in this programme were satisfied in terms of free services and compensation. However, despite the free services provided by SBAs and suitable incentives for poor pregnant women, the programme seemed to be unattractive for pregnant women who lived in some remote areas that were very far from health facilities as the financial benefits were insufficient due to the higher costs of paying for commutes to the health facilities.

This study indicated that only one additional MW was allocated to Yedarshey Township after the initiation of the programme despite significant increased service burden to the health facilities. Nevertheless, MW informants maintained that they could cope with the additional workload since their clients rarely visited at the same time. The MWs affirmed that their current capacity had not yet reached the maximum limit. While the current workload of the MWs in particular areas varied largely between 0 and 12 deliveries, it was found that one MW could provide up to 20 deliveries per month, and most of them currently provided an average of 3.5 deliveries per month. Therefore, the MWs would be able to accommodate the increasing demand generated by the MCHVS. Some informants argued that MWs in Myanmar had to spend a lot of time in meetings in the township and attending training programmes, and if they had been excused from these activities, more services could be delivered.

Pregnant women, voucher distributors, and MWs believed that ANC and delivery by AMWs and TBAs might decrease as only those who were not eligible for the vouchers continued to seek care from them. Key informants also argued that this change in practice would not have a significantly negative effect on their income because prior to the MCHVS’s launch, AMWs and TBAs did not earn much money by providing services to clients in the poorest group. In fact, poor pregnant women sometimes paid AMWs and TBAs with agricultural products as well as natural products from the forest instead of cash. At the same time, AMWs and TBAs generally had more than one job or had an alternative major source of income compared to delivery service from which they earned small amounts of money. It is anticipated that some eligible pregnant women who live in remote areas where MCH services are unavailable will still use the services of AMWs and TBAs.

## Discussion

This evaluation was mainly focused on two major issues, implementation process and primary outcomes of the programme. Overall, the MCHVS was implemented following the guidelines and recommendations from the previous studies [18]. It was confirmed that the financial mechanism of the pilot programme satisfied both providers and beneficiaries, however, there is still some room for improvement.

Even though communication between MWs and beneficiaries was good, the most important message of the programme, i.e. health benefits, was not communicated well. Financial incentives, which were the perceived benefits, may not make the programme sustainable, which is evident in other settings; for example, in Bangladesh cash benefits did not help beneficiaries directly [20]. In this study, some pregnant women who lived in remote areas that were very far from the health facilities lost their motivation in using vouchers. Thus, the health benefits of the programme and communicating them to the beneficiaries should be reiterated to all voucher distributors. Moreover, internal communication between the voucher distributors and administrative body is also important. The results show that there were some vouchers left even though the number of vouchers that were distributed was less than the number of registered beneficiaries. This might be because of a lack of internal communication, which led to a baseless rumour about the scheme.

In terms of voucher distributors, the problem of recruiting non-MWs to be voucher distributors should be set as a high priority that needs to be addressed. Having only MWs as voucher distributors could create inadequate voucher distribution because MWs seemed likely to give vouchers to those who visited them at health facilities [21]. Voluntary distributors can help increasing access to services of pregnant women and their babies [22]. For example, in Cambodia, the distributors had different backgrounds, such as village volunteers, commune council members, women representatives, community-based organisation, community leaders, and other community representatives, and distributed a number of vouchers to the beneficiaries [23].

Although the non-MW suggested including financial incentives, this issue needs to be considered cautiously as negative consequences could occur. To some degree, these requests may raise costs of the programme but cannot increase coverage of vouchers and services as expected. Furthermore, experiences about roles, efficiency, and effectiveness of assigning non-MWs to be voucher distributors from other settings, especially low- and middle-income countries, are important to research for the programme. In some cases, problems of distributing vouchers by voluntary distributors could be due to a lack of trust in the communities or an inability to get those beneficiaries who lived in remote areas enrolled due to transportation problems [22].

For the beneficiary identification process, inappropriate criteria were found to be major concerns. This issue also happened in other settings [24]. Therefore, the criteria that were used to screen beneficiaries may need to be revised to ensure that those who really need the services will not be excluded from the programme. Additionally, in order to identify and distribute vouchers, voucher distributors must be very careful in some areas as they can create stigmatisation to the beneficiaries. This is because the policies made people feel ashamed for being classified as vulnerable and strictly dependent on support from the government or others [25–27]. Building up knowledge about maternal and child health both on individual and community levels could improve awareness and behaviour on seeking care and health outcomes and reduce stigmatization [28].

One impediment to engage poor pregnant women who live in remote area into the MCHVS was insufficient compensation, which was not attractive enough for them to come and receive care at health facilities. In order to address this issue, there is an example from the voucher programme in Cambodia that offered beneficiaries financial compensation which depended on the distance between their houses and health facilities [29]; this was also similarly suggested for the cash incentive programme in Nepal [30]. Nonetheless, to implement this approach, the management team needs to carefully consider the amount of money that can be reimbursed. In addition, the Cambodia voucher programme study showed that some beneficiaries were uncertain that they could fully reimburse the transportation fee, indicating distrust in the programme.

This study indicates that after 7 months of programme implementation, new mothers and the newborns received essential health care from skilled health personnel. This may lead to an improvement of health outcomes, for example, reducing maternal and neonatal mortality, in the future. Evidence shows that receiving care, particularly delivery, from skilled health personnel can bring about a significant reduction of maternal and neonatal mortalities [31], although the direct link between maternal and child health outcomes and the voucher utilisation is difficult to obtain due to confounding factors of other co-interventions that were implemented and might be overlapped with the MCHVS. Importantly, the actual factors that associate low and inappropriate voucher distribution and utilisation should be sought out to make a proper and contextualised resolution for the programme.

During the period of programme design, provider capacities and quality of the services were not taken into account, although the increased workload of SBAs after implementing the programme, which might affect the quality of services, could be estimated. Therefore, these two issues were not included in this evaluation. However, the information from SSIs indicated that MWs are able to handle more than their workload during the early stage of implementation. So, an estimation of the workforce (doctors, nurses, and MWs) should be examined to support the probable increase in number of beneficiaries in the later phases of the programme, as well as systematic monitoring and evaluation systems on quality of services regarding the increasing workload, as it was found that quality services can be influenced by low satisfaction of health providers [32], especially when work burden increases [33].

While the strength of qualitative research is the ability to have two-way communication between researchers and key informants in order to gain an in-depth understanding of complex issues and contexts that influence those issues, the major challenge of this study was the qualitative data collection as a result of language barriers between the evaluators and key informants, especially in SSIs in which interpretation was needed for the evaluators to be understood in both specific topics and new topics that emerged during the interviews. The team observed that, in this evaluation, appointing translators in the interviews had some flaws, for example, missing points in the conversation, changes in meaning as a result of the translation, or pauses in interviews that resulted in missed information. In addition, the interviewees may have been more hesitant to be candid in their answers due to social hierarchy, such as having government officers from the MoH, Myanmar as translators when a majority of key informants were lay people. This could create biases in information because key informants were reluctant to give the certain types of information. Some evidence points to these two challenges, which can create low quality data, partly concealed data, or inaccurate data [34–37].

## Conclusions

Although the relationship between the MCHVS and health outcomes has not taken into in this evaluation yet, the trend of MCH services access by skilled health professional in Yedarshey Township seems to be promising towards the attitudes of providers and beneficiaries and services utilisation under the programme. The next step of the programme should be planned very well regarding several impediments that were found in this evaluation, viz. external and internal programme communication, voluntary voucher distributor recruitment, incentive and support for voucher distributors, beneficiary screening criteria, and approaches to access to the services of pregnant women who lived in remote area.

## Abbreviations

AMWs: Auxiliary midwives
ANC: Antenatal care
MCH: Maternal and child health
MCHVS: Maternal and child health voucher scheme
MMK: Myanmar Kyats
MoH: Ministry of Health
MWs: Midwives
PNC: Post-natal care
RHCs: Rural health centres
SBAs: Skilled birth attendants
SSIs: Semi-structured interviews
TBAs: Traditional birth attendants
WHO: World Health Organization
